# Management of fetal umbilical vein varix: a survey-based analysis of current clinical practice

**DOI:** 10.1007/s00404-025-08260-8

**Published:** 2026-01-14

**Authors:** Rachelle Fraenkel Merzbach, Reut Rotem, Dan V. Valsky, Hen Y. Sela, Simcha Yagel, Misgav Rottenstreich

**Affiliations:** 1 Division of Obstetrics and Gynecology, Hadassah-Hebrew University Medical Center, Mt Scopus, Jerusalem, Israel; 2 Department of Obstetrics and Gynecology, Shaare Zedek Medical Center, Affiliated with the Hebrew University School of Medicine, Jerusalem, Israel

**Keywords:** Fetal intra-abdominal umbilical vein varix, Prenatal diagnosis, Risk stratification, Antenatal surveillance, Delivery timing

## Abstract

**Objective:**

To evaluate current diagnostic criteria, risk stratification, and management practices for fetal intra-abdominal umbilical vein varix (FIUVV) among maternal–fetal medicine and obstetric ultrasound specialists in Israel.

**Methods:**

A national web-based survey was distributed to specialists, collecting data on diagnostic parameters, classification of severity, surveillance protocols, and delivery timing recommendations. Responses were analyzed descriptively.

**Results:**

45 specialists (85% response) from 18 centers participated. Diagnosis was most commonly based on FIUVV diameter, with 9 mm the most frequent cutoff, though substantial variation in criteria existed. Severity assessment relied mainly on vessel diameter, flow abnormalities, and thrombus presence. Most respondents recommended detailed anatomical survey and fetal echocardiography. Surveillance and delivery timing recommendations were tailored to risk level, with earlier induction and increased surveillance favored in high-risk cases. However, considerable variability existed among respondents regarding both the frequency of monitoring and the timing of delivery**.**

**Conclusions:**

Considerable heterogeneity exists in FIUVV diagnosis and management in Israel. Consensus definitions and standardized protocols are needed to improve clinical care and enable comparative research.

**Supplementary Information:**

The online version contains supplementary material available at 10.1007/s00404-025-08260-8.

## Take-home message

**Table:** 

Despite general aggrement that isolaated FIUVV carries a favorable prognosis, substantial variability in diagnostic criteria and management strategies persists, underscoring the need for consensus-based guidelines.	

## Introduction

Fetal intra-abdominal umbilical vein varix (FIUVV) refers to focal dilatation of the intra-abdominal portion of the umbilical vein, identified through prenatal ultrasonography. This vascular anomaly is relatively rare, affecting 0.4–2.8 per 1000 pregnancies [1, 2]. The normal umbilical vein diameter exhibits a progressive increase throughout gestation, ranging from 2 mm at 15 weeks to 7–8 mm at term [3].

Diagnostic criteria for FIUVV vary, with definitions based on absolute diameter, deviation from gestational norms, or relative enlargement compared to adjacent vessel segments, making standardized diagnosis and management more challenging [3–6].

Early studies suggested high fetal mortality rates associated with FIUVV, with some estimates reaching up to 40% [3, 7]. However, contemporary research has demonstrated considerably lower mortality rates of less than 1% [2]. Recent meta-analyses suggested that isolated FIUVV, i.e., in the absence of additional anatomical abnormalities—does not significantly increase the risk of IUFD or chromosomal abnormalities, though it may be associated with fetal growth restriction (FGR) [8, 9].

Despite the accumulating evidence on the implications of FIUVV, there is still no clear consensus in the literature regarding diagnostic criteria, optimal pregnancy surveillance, and management protocols [8]. This variation in management strategies may contribute to different clinical practices, both between and within medical institutions, which could influence patient care and outcomes.

The aim of this survey was to evaluate the current practice in respect of diagnosis and management of FIUVV among maternal–fetal medicine (MFM) specialists and obstetric ultrasound experts across medical centers in Israel.

## Materials and methods

### Study design and setting

A cross-sectional, multi-center national survey was conducted between 2023 and 2024 among MFM and obstetric ultrasound specialists in Israel. The survey targeted expert physicians working in obstetric units across 20 university-affiliated medical centers.

### Ethical considerations

As the study did not involve direct patient participation, approval from the internal review board was not deemed necessary.

### Survey development

The survey was developed by two researchers (RD, MR), both specialists in obstetrics and gynecology, drawing on literature review and expert input. The questionnaire was designed to capture current clinical practices regarding FIUVV, including (Appendix S1):

- Definitions and diagnostic criteria
- Severity grading
- Recommendations for additional diagnostic workup
- Surveillance protocols
- Timing and mode of delivery

Each item included categorical response options and a field to allow clinicians to share their individual perspectives and experiences. The survey was pilot-tested for clarity and relevance among a small group of specialists before distribution.

### Participant recruitment

Eligible participants were MFM and obstetric ultrasound specialists actively involved in the diagnosis and management of FIUVV, listed based on professional networks. Invitations were distributed via email, with a secure link to the electronic survey (Google Forms; see Appendix S1). Participation was voluntary, and responses were collected anonymously. Data transmission between respondents’ devices and Google’s servers is encrypted using HTTPS, and responses are stored on Google’s secure servers with encryption at rest. Access to raw data was restricted to the study investigators through account-based permissions.

### Data collection

The survey remained open for a period of 3 months (November 2023–February 2024), with two reminder emails sent to maximize response rates. Data collected included respondent demographics (years of experience, institutional affiliation), as well as detailed responses to clinical practice questions.

### Data analysis

Survey data were exported from Google Forms and analyzed using SPSS software (Version 23, IBM, NYC, USA). Descriptive statistics were used to summarize responses, including frequencies and percentages for categorical variables and medians with interquartile ranges (IQR) for continuous variables.

## Quality assurance

To ensure data integrity, responses were reviewed for completeness and consistency. Duplicate or incomplete submissions were excluded from the final analysis.

## Results

### Survey response and participants

A total of 53 specialists from university-affiliated medical centers with delivery units were invited to participate in the survey, of whom 45 responded, yielding a response rate of 85%. The responses were obtained from 18 medical centers in Israel, representing 66.6% of the 27 medical centers nationwide. The participants had a median of 16 years of experience in obstetrics and gynecology; 53% were obstetric ultrasound specialists, and 47% were MFM specialists; 62% were male and 38% were female.

#### Definition and criteria for diagnosis

With regard to the definition of FIUVV, most respondents (61.4%) reported using the intra-abdominal umbilical vein diameter at the cord insertion site as the sole diagnostic parameter. A smaller proportion (36.4%) used both the diameter of the umbilical vein and the ratio between the intra and extra-abdominal portions of the umbilical vein, and only one specialist relied on the ratio alone for diagnosis.

##### Criteria for diagnosis

*Intra-abdominal diameter of the umbilical vein*: Most of the respondents (63%) reported using a diagnostic cutoff of 9 mm, followed by 10 mm (30%), 8 mm (5%), and 11 mm (2%)*.*

*Ratio between the diameter of the intra and extra-abdominal umbilical vein:* Among those who use this ratio as a diagnostic criterion (*n* = 25), 96% reported a cutoff of ≥ 1.5, whereas only one (4%) reported a cutoff of 2.

#### Severity of FIUVV

Most respondents (73%) perceived FIUVV as a condition that can manifest in varying degrees of severity.

**Fig. 1:**
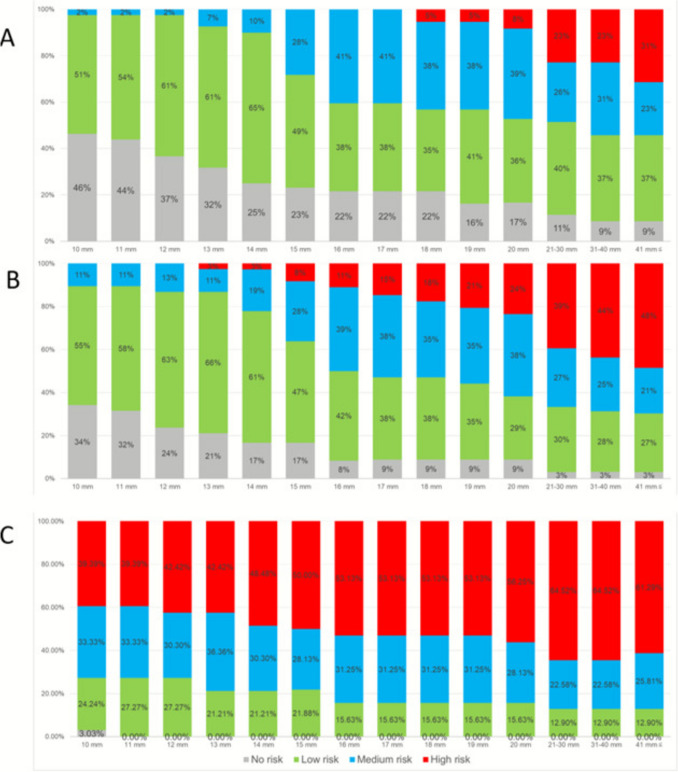
The severity of FIUVV by the diameter of the umbilical vein and pattern of flow or the presence of a clot within the FIUVV. **a** linear flow within the FIUVV. **b** turbulent flow within the FIUVV. **c** presence of a clot within the FIUVV

*Severity by diameter, flow pattern and presence of a clot*: Figure 1a illustrates specialists’ assessments of the severity of isolated FIUVV (in the absence of other anatomical or growth abnormalities) based on diameter with linear flow. Most respondents associated any diameter with some risk of perinatal mortality, and at diameters ≥ 21 mm, approximately 50% rated the risk as moderate to high. With turbulent flow, more than half considered a diameter > 16 mm indicative of moderate to high risk (Fig. 1b). The presence of a clot was perceived as a substantial risk factor for perinatal mortality, with nearly 75% rating the risk as moderate or high regardless of diameter. When a clot was present and the diameter exceeded 15 mm, most classified the case as high risk for perinatal death (Fig. 1c).

*The severity of FIUVV by the ratio of the intra and extra-abdominal umbilical vein*: Among respondents using the ratio as a diagnostic criterion (*n* = 25), 64% considered a ratio above 1.5 indicative of a high perinatal mortality risk, 27% used a cutoff of 2, and 9% selected > 2 as their threshold for high risk.

#### Diagnosis

**Fig. 2:**
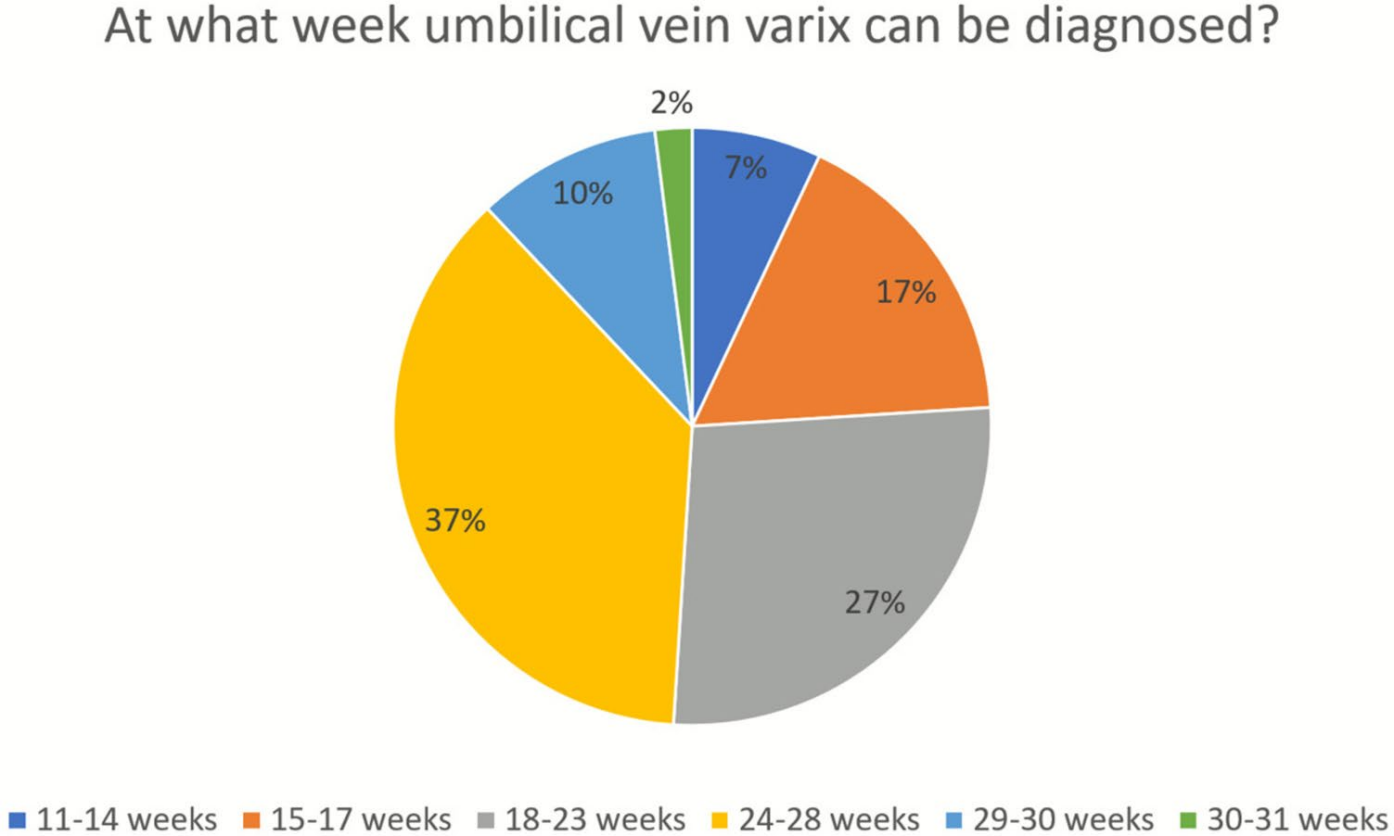
Earliest gestational age at which the diagnosis of FIUVV can be made

*Time of diagnosis*: Nearly half of respondents (49%) believed FIUVV can only be diagnosed in the third trimester, while 44% considered it detectable as early as the second trimester (Fig. 2).Most specialists (68%) thought the time of diagnosis does not influence FIUVV severity, whereas 32% believed earlier diagnosis is associated with higher perinatal mortality risk.

#### Evaluation and surveillance of FIUVV

**Fig. 3:**
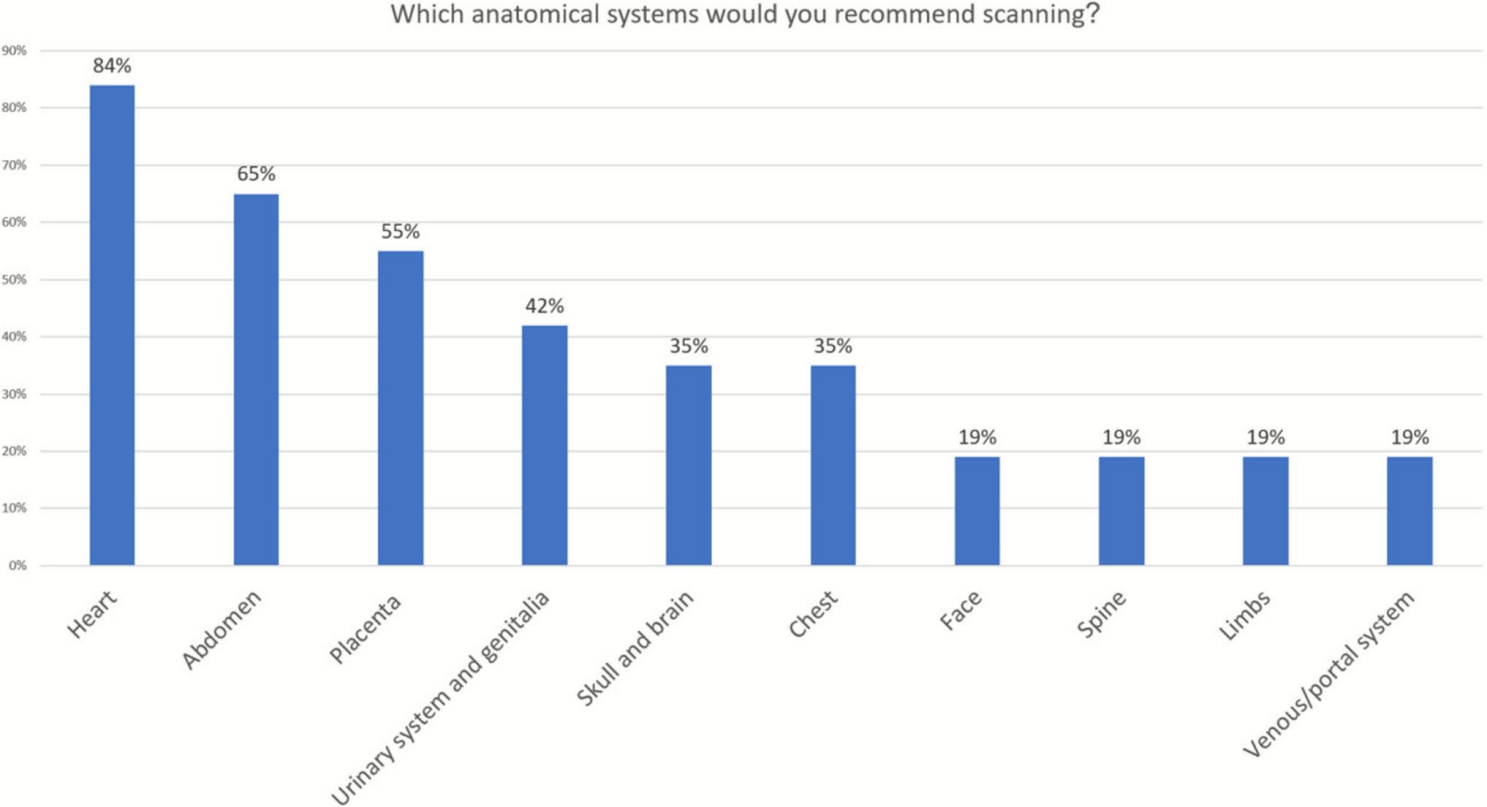
Suggestions regarding organ systems to be evaluated in a detailed anatomic scan

*Evaluation:* Most responders (87%) believed further investigation is warranted following the diagnosis of FIUVV. The majority recommended fetal echocardiography (83%), a detailed anatomical survey (69%), and fetal growth monitoring (67%), while 49% suggested a genetic consultant and testing. Figure 3 illustrates the systems and organs that were suggested to be evaluated in the detailed anatomical survey, including the heart (84%), abdomen (65%), placenta (55%), and urinary system and genitalia (42%).

**Fig. 4:**
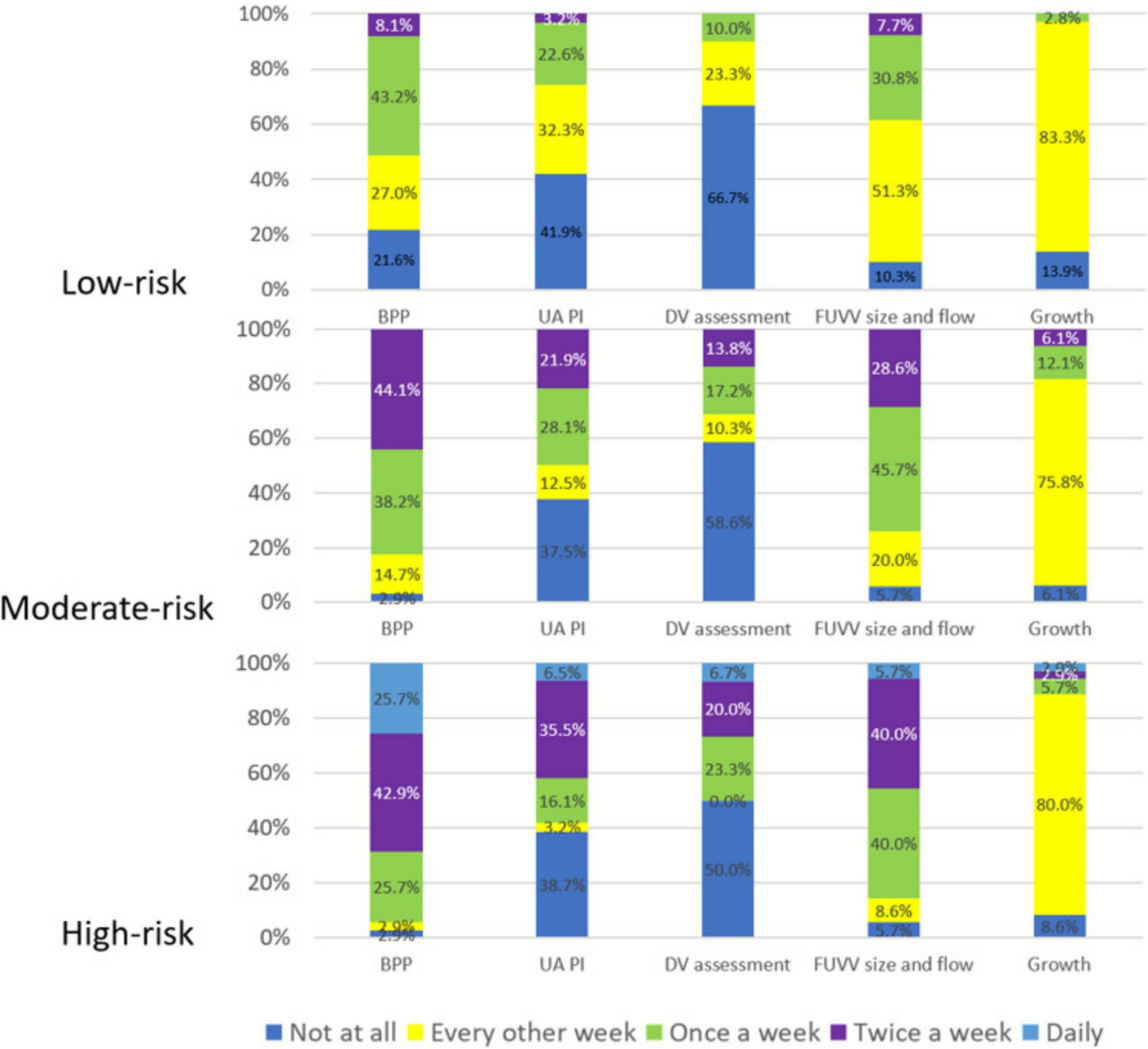
Specialists’ answers regarding the necessary follow-up following a diagnosis of FIUVV

*Surveillance*: As shown in Fig. 4, specialists tailored follow-up based on the perceived risk level of FIUVV. For low-risk FIUVV, most recommended fetal heart rate monitoring, evaluation of varix size and flow, and umbilical artery pulsatility index every 1 to 2 weeks, alongside fetal growth assessment at 2-week intervals. In moderate-risk situations, recommended surveillance increased to once or twice weekly, and for high-risk cases, approximately 67% advised surveillance either daily or twice weekly. Across all risk levels, the majority supported the inclusion of ductus venosus flow assessment.

*Hospital admission:* Most respondents (67%) did not consider hospital admission necessary in FIUVV cases, while 28% believed it was indicated in high-risk cases.

*Indication for termination of pregnancy*: None of the specialists considered FIUVV, regardless of severity, as an indication for offering termination of pregnancy.

#### Delivery

**Fig. 5:**
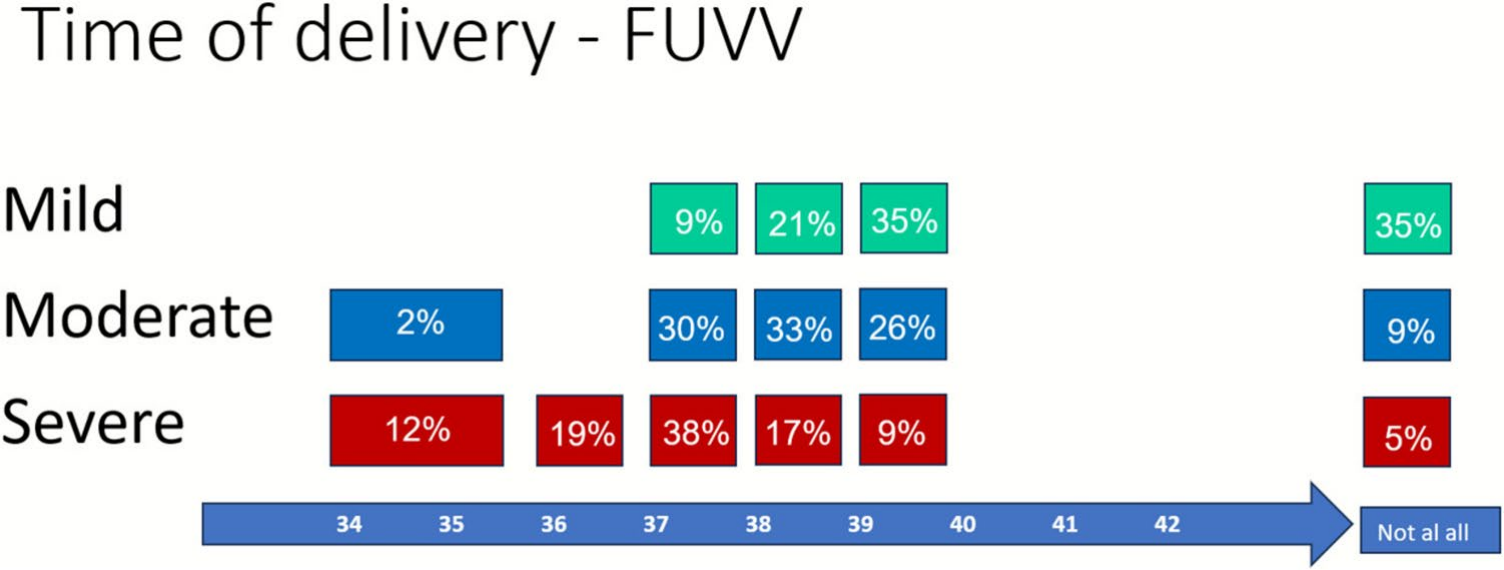
Timing of delivery

*Timing for delivery:* Figure 5 presents the specialists’ recommendations for timing of delivery based on FIUVV severity. For low-risk FIUVV, the majority (75%) recommended induction of labor at term (9% at 37 weeks, 21% at 38 weeks, and 35% at 39 weeks), 35% believed there is no need for timed delivery at any stage, and none recommended induction before term. For moderate-risk FIUVV, nearly all respondents (89%) advised induction at term (30% at 37 weeks, 33% at 38 weeks, and 26% at 39 weeks), 9% believed there is no need for timed delivery, and one respondent (2%) supported induction before term. In high-risk FIUVV cases, 31% recommended induction before term (12% at 34–35 weeks and 19% at 36 weeks), another 38% chose 37 weeks, and 26% preferred induction between 38 and 39 weeks. Still, 5% responded that there is no need to time the delivery.

*Mode of delivery*: The vast majority of respondents (96%) supported trial of labor in the absence of other indications for cesarean delivery.

## Discussion

This national survey of maternal–fetal medicine and obstetric ultrasound specialists in Israel highlights substantial variability in the diagnosis, risk stratification, and management of FIUVV. Although most clinicians rely primarily on intra-abdominal umbilical vein diameter, with 9 mm as the most frequently used cutoff, there is no uniform criterion for defining FIUVV or for determining disease severity. Despite this heterogeneity, a shared understanding exists that isolated FIUVV generally carries a favorable prognosis and does not in itself warrant cesarean delivery or pregnancy termination.

## Results in the context of what is known

Our findings are consistent with the wide variation described in previous reports and demonstrate that no single, universally adopted criterion for the diagnosis of FIUVV exists in clinical practice. The majority of respondents reported using the intra-abdominal diameter of the umbilical vein immediately after cord insertion as the primary diagnostic parameter, while a significant proportion incorporate both absolute diameter and the ratio between the intra and extra-abdominal portions of the umbilical vein. This diversity underscores the absence of standardized diagnostic definitions and the challenge it poses for both clinical practice and research comparability, as in recent reviews and meta-analyses, both thresholds of ≥ 9 mm, a diameter exceeding 2 standard deviations above the mean for gestational age, or a > 50% increase compared to the non-dilated segment have been variably employed [2–6, 8–16]. Importantly, while many respondents in our survey equated larger varix size with increased risk, this is not aligned with the published literature, as no such classifications have been reported, and available evidence suggests that associated findings such as turbulent flow, thrombus formation, structural anomalies, or fetal growth restriction are stronger predictors of adverse outcomes than diameter alone [8, 10, 11].

In line with prior studies, most specialists recommended comprehensive fetal evaluation following FIUVV detection, including detailed anatomical survey and echocardiography together with genetic counseling when additional findings are present. This approach reflects the reported 10–20% incidence of additional anomalies [2, 9, 11, 14, 17] with cardiac, genitourinary, and central nervous system defects being the most frequent ones, as well as up to 10% rate of chromosomal abnormalities [8, 10, 11]. These data emphasize the importance of thorough assessment rather than reliance on vessel measurements alone to guide counseling and surveillance intensity.

## Clinical implications

Earlier studies reported a higher incidence of non-isolated FIUVV [16, 18], suggesting a potential temporal shift in detection or classification. Both the literature and our survey emphasize the importance of comprehensive fetal evaluation to guide prognosis.

There is considerable variation in surveillance protocols for FGR and distress among respondents, reflecting the lack of consensus in the literature. Reported rates of FGR in FIUVV cases range from 3.7% to 7.6%, with higher prevalence among non-isolated cases [8–10, 15]. In addition, while early reports suggested fetal mortality rates as high as 40% [3], more recent studies and meta-analyses indicated that isolated FIUVV carries IUFD risk comparable to the general population [8, 9, 11, 13, 15, 16]. Therefore, intensified surveillance may be more appropriate for non-isolated cases. However, it is essential to point out that in both meta-analyses showing no increase in IUFD rates in isolated FIUVV, the mean gestational age at delivery was around 37–38 weeks [8, 9]. Prudence is therefore still warranted when making recommendations regarding the management of these pregnancies beyond the aforementioned gestational weeks.

Management of cases complicated by turbulent flow or thrombus remains an area of clinical uncertainty, as reflected in both our survey and the literature. Current literature suggests that while turbulent flow—and especially thrombus formation—warrant increased surveillance, management should be individualized based on gestational age, additional risk factors, and the capacity for close monitoring [9–11, 19]. When thrombus is identified, most clinicians favor delivery due to the theoretical risk of sudden IUFD. However, in selected early gestational cases, expectant management with intensive monitoring may be considered following thorough counseling regarding potential risks [8].

## Future directions

This study serves as a call for action to our professional community, which values evidence-based medicine, personalized care, and shared decision-making. Future studies should aim to delineate criteria for diagnosis and validate objective severity criteria for FIUVV and assess their correlation with perinatal outcomes. Prospective cohort studies evaluating outcomes by diameter, flow characteristics, and thrombus presence are essential to refine risk stratifications. In parallel, assessing the cost-effectiveness and clinical impact of various surveillance protocols may help develop standardized yet individualized management pathways. Given the lack of evidence for increased IUFD in isolated FIUVV, further research should focus on identifying subsets of patients who may benefit from more intensive monitoring or early delivery.

## Strengths and limitations

The strengths of this study include its nationwide scope, high response rate from major academic centers, and focus on real-world clinical decision-making by experts in obstetric ultrasound and MFM. To our knowledge, it is the first study to systematically assess clinical management practices of FIUVV on a national scale. However, limitations include its reliance on self-reported practices, which may not always reflect actual clinical behavior. Furthermore, the survey was limited to a single country, and therefore results should be retested in other healthcare systems prior to adoption of our findings and conclusions.

## Conclusions

In summary, this study reveals both areas of consensus and substantial heterogeneity in the diagnosis and management of FIUVV. The absence of standardized definitions and protocols hampers uniform clinical care and comparative research. Our findings reinforce the urgent need for international collaboration to develop evidence-based diagnostic criteria and management pathways that optimize outcomes for pregnancies complicated by FIUVV.

## Supplementary Information

Below is the link to the electronic supplementary material.

Supplementary file1 (DOCX 36 KB)

## Data Availability

The de-identified survey dataset, codebook, and analysis syntax are available from the corresponding author on reasonable request.

## Declarations

### AI use statement

Artificial intelligence tools were used only for language editing and organization of the text. All scientific content, data interpretation, and references were generated and verified exclusively by the authors.

## References

[1] Beraud E, Rozel C, Milon J, Darnault P (2015) Umbilical vein varix: importance of ante- and post-natal monitoring by ultrasound. Diagn Interv Imaging 96(1):21–26. 10.1016/j.diii.2014.01.009 24631035 10.1016/j.diii.2014.01.009

[2] Lee SW, Kim MY, Kim JE, Chung JH, Lee HJ, Yoon JY (2014) Clinical characteristics and outcomes of antenatal fetal intra-abdominal umbilical vein varix detection. Obstet Gynecol Sci 57(3):181 24883288 10.5468/ogs.2014.57.3.181 PMC4038683

[3] Mahony BS, McGahan JP, Nyberg DA, Reisner DP (1992) Varix of the fetal intra-abdominal umbilical vein: comparison with normal. J Ultrasound Med 11(2):73–76 1560496 10.7863/jum.1992.11.2.73

[4] Woodward PJ, Kennedy A, Sohaey R (2016) Diagnostic imaging: obstetrics, 3rd edn. Elsevier, Philadelphia, pp 826–827. Available from: https://shop.elsevier.com/books/diagnostic-imaging-obstetrics/woodward/978-0-323-39256-3

[5] Sepulveda W, Mackenna A, Sanchez J, Corral E, Carstens E (1998) Fetal prognosis in varix of the intrafetal umbilical vein. J Ultrasound Med 17(3):171–175 9514169 10.7863/jum.1998.17.3.171

[6] Melcer Y, Ben-Ami I, Wiener Y, Livne A, Herman A, Maymon R (2013) Long-term outcomes of children with umbilical vein varix diagnosed prenatally. Prenat Diagn 33(5):492–496 23529797 10.1002/pd.4098

[7] Valsky DV, Rosenak D, Hochner-Celnikier D, Porat S, Yagel S (2004) Adverse outcome of isolated fetal intra-abdominal umbilical vein varix despite close monitoring. Prenat Diagn 24(6):451–454 15229845 10.1002/pd.897

[8] di Pasquo E, Kuleva M, O’Gorman N, Ville Y, Salomon LJ (2018) Fetal intra-abdominal umbilical vein varix: retrospective cohort study and systematic review and meta-analysis. Ultrasound Obstet Gynecol 51(5):580–585 28876490 10.1002/uog.18895

[9] Koorn I, Heinrich H, Nelissen A, Denswil N, Linskens IH, Jansen CHJR et al (2024) Isolated fetal umbilical vein varix and the association with intrauterine fetal death and fetal growth restriction: a systematic review, meta-analysis, and nested retrospective cohort study. Prenat Diagn 44(5):595–613 38502055 10.1002/pd.6538

[10] Ghahremani T, Britt AB, Ray E, Jones A, Du R, Magann EF (2025) Perinatal outcomes and management of umbilical vein varix: a comprehensive review of 392 cases. J Clin Med 14(2):1–14 10.3390/jcm14020441 PMC11765959 39860448

[11] Novoa V, Shazly S, Ibirogba ER, Sutton L, Tonni G, Prefumo F et al (2021) Perinatal outcomes of fetal intra-abdominal umbilical vein varix: a multicenter cohort study. J Matern Fetal Neonatal Med 34(20):3393–3396. 10.1080/14767058.2019.1685969 31736416 10.1080/14767058.2019.1685969

[12] Nyberg DA, McGahan JP, Pretorius DH, Pilu G (2002) Chapter 4—The placenta, umbilical cord, and membranes. Diagnostic imaging of fetal anomalies, 2nd edn. Lippincott Williams & Wilkins, Philadelphia, pp 114–115

[13] Weissmann-Brenner A, J. Simchen M, Moran O, Kassif E, Achiron RZY (2009) Isolated fetal umbilical vein varix—prenatal sonographic diagnosis and suggested management. Prenat Diagn 29(3):229–233 19177454 10.1002/pd.2219

[14] Bas-Lando M, Rabinowitz R, Samueloff A, Latinsky B, Schimmel MS, Chen O et al (2013) The prenatal diagnosis of isolated fetal varix of the intra-abdominal umbilical vein is associated with favorable neonatal outcome at term: a case series. Arch Gynecol Obstet 288(1):33–39 23389248 10.1007/s00404-013-2743-x

[15] Quinn K, Streich-Tilles T, Wade J, Cartledge J, Denney JM (2024) Umbilical vein varix ultrasound characteristics and perinatal outcomes: a retrospective cohort study. Arch Obstet Gynaecol 5(2):78–86

[16] Byers BD, Goharkhay N, Mateus J, Ward KK, Munn MB, Wen TS (2009) Pregnancy outcome after ultrasound diagnosis of fetal intra-abdominal umbilical vein varix. Ultrasound Obstet Gynecol 33(3):282–286 19115263 10.1002/uog.6233

[17] Mankuta D, Nadjari MPG (2011) Isolated fetal intra-abdominal umbilical vein varix: clinical importance and recommendations. J Ultrasound Med 30(2):273–276 21266567 10.7863/jum.2011.30.2.273

[18] Fung TY, Leung TN, Leung TY, Lau TK (2005) Fetal intra-abdominal umbilical vein varix: what is the clinical significance? Ultrasound Obstet Gynecol 25(2):149–154 15685644 10.1002/uog.1815

[19] Zhan J, Wang D, Luo C, Bi H (2024) Umbilical vascular thromboembolism: high-risk factors, diagnosis, management, and pregnancy outcomes: a scoping review. Ther Clin Risk Manag 20:597–610 39263225 10.2147/TCRM.S478593 PMC11389714

