# Supplementary material for: Management of fetal umbilical vein varix: a survey-based analysis of current clinical practice
Source: Arch Gynecol Obstet. 2026 Jan 14;313(1):42. doi: 10.1007/s00404-025-08260-8 (PMC12804326; doi:10.1007/s00404-025-08260-8)
Supplement: Supplementary file 1 — Supplementary file1 (DOCX 36 KB) [file 404_2025_8260_MOESM1_ESM.docx]

Appendix S1-Questionnaire on the Management of Umbilical Vein Varix

Clarification for respondents prior to completing the questionnaire:
This survey is administered via Google Forms, an open-access platform. Please note the potential risk of data exposure.

# Definition of Umbilical Vein Varix (UVV)

1. According to which criteria would you establish the diagnosis of intra-abdominal Umbilical Vein Varix?
☐ Based on the maximal diameter of the intra-abdominal umbilical vein at the point of cord insertion into the fetal abdomen, prior to entry into the portal system
☐ Based on the ratio between the diameter of the dilated segment of the intra-abdominal umbilical vein and the diameter of its extra-abdominal portion prior to insertion into the umbilicus
☐ Both of the above
☐ Other: __________

2. If the diagnosis is based on maximal diameter at insertion, above what threshold would you define UVV?
☐ 7 mm ☐ 8 mm ☐ 9 mm ☐ 10 mm ☐ 11 mm ☐ 12 mm ☐ 13 mm ☐ 14 mm ☐ 15 mm ☐ 16 mm ☐ 17 mm ☐ 18 mm ☐ 19 mm ☐ 20 mm ☐ Other: ____

3. If the diagnosis is based on the ratio between the dilated and extra-abdominal segments, what ratio defines UVV?
☐ 1.1× ☐ 1.2× ☐ 1.3× ☐ 1.4× ☐ 1.5× ☐ 1.6× ☐ 1.7× ☐ 1.8× ☐ 1.9× ☐ 2× ☐ Other: ____

# Severity Grading of UVV

4. Does Umbilical Vein Varix have severity grades?
☐ No ☐ Yes

5. If yes, in isolated UVV without anomalies, estimate IUFD risk at each diameter (linear flow).

| High risk for IUFD | Moderate risk for IUFD | Mild risk for IUFD | No risk for IUFD |  |
| --- | --- | --- | --- | --- |
|  |  |  |  | 10 mm |
|  |  |  |  | 11 mm |
|  |  |  |  | 12 mm |
|  |  |  |  | 13 mm |
|  |  |  |  | 14 mm |
|  |  |  |  | 15 mm |
|  |  |  |  | 16 mm |
|  |  |  |  | 17 mm |
|  |  |  |  | 18 mm |
|  |  |  |  | 19 mm |
|  |  |  |  | 20 mm |
|  |  |  |  | 21-30 mm |
|  |  |  |  | 31-40 mm |
|  |  |  |  | 41 ומעלה |

6. In isolated UVV, estimate IUFD risk at each diameter (turbulent flow).

| High risk for IUFD | Moderate risk for IUFD | Mild risk for IUFD | No risk for IUFD |  |
| --- | --- | --- | --- | --- |
|  |  |  |  | 10 mm |
|  |  |  |  | 11 mm |
|  |  |  |  | 12 mm |
|  |  |  |  | 13 mm |
|  |  |  |  | 14 mm |
|  |  |  |  | 15 mm |
|  |  |  |  | 16 mm |
|  |  |  |  | 17 mm |
|  |  |  |  | 18 mm |
|  |  |  |  | 19 mm |
|  |  |  |  | 20 mm |
|  |  |  |  | 21-30 mm |
|  |  |  |  | 31-40 mm |
|  |  |  |  | 41 ומעלה |

7. In isolated UVV, estimate IUFD risk at each diameter (suspected thrombosis).

| High risk for IUFD | Moderate risk for IUFD | Mild risk for IUFD | No risk for IUFD |  |
| --- | --- | --- | --- | --- |
|  |  |  |  | 10 mm |
|  |  |  |  | 11 mm |
|  |  |  |  | 12 mm |
|  |  |  |  | 13 mm |
|  |  |  |  | 14 mm |
|  |  |  |  | 15 mm |
|  |  |  |  | 16 mm |
|  |  |  |  | 17 mm |
|  |  |  |  | 18 mm |
|  |  |  |  | 19 mm |
|  |  |  |  | 20 mm |
|  |  |  |  | 21-30 mm |
|  |  |  |  | 31-40 mm |
|  |  |  |  | 41 ומעלה |

8. If severity grading is based on ratio, from what ratio is UVV considered severe?
☐ Not determinant ☐ 1.1× ☐ 1.2× ☐ 1.3× ☐ 1.4× ☐ 1.5× ☐ 1.6× ☐ 1.7× ☐ 1.8× ☐ 1.9× ☐ 2× ☐ >2× ☐ Other: ____

# Timing of Diagnosis

9. From which gestational age is UVV diagnosable?
☐ 11–14 ☐ 15–17 ☐ 18–23 ☐ 24–28 ☐ 29–30 ☐ 30–31 ☐ 32–33 ☐ 34–35 ☐ 36 ☐ 37 ☐ 38 ☐ 39 weeks

10. Does gestational age at diagnosis influence severity?
☐ No ☐ Yes, earlier diagnosis = more severe ☐ Yes, later diagnosis = more severe

# Termination of Pregnancy

11. In severe UVV diagnosed prior to viability, should TOP be considered to prevent extreme prematurity?
☐ Yes ☐ No

# Recommended Work-up

12. In UVV, would you recommend further evaluation?
☐ Yes ☐ No

13. If yes, which investigations?
☐ Targeted anomaly scan ☐ Fetal echocardiography ☐ Genetic counseling ± amniocentesis ☐ Growth surveillance ☐ Other: ____

14. If targeted scan, which systems should be evaluated?
☐ Skull/Brain ☐ Face ☐ Thorax ☐ Heart ☐ Abdomen ☐ Spine ☐ Limbs ☐ Placenta ☐ Urinary/Genital ☐ Other: ____

# Surveillance

15. For UVV of mild, moderate, or severe grade, what frequency of monitoring do you recommend for key parameters?

15. 1 For Mild grade

| Not at all | Once in two weeks | Once a week | Twice a week | Once a day |  |
| --- | --- | --- | --- | --- | --- |
|  |  |  |  |  | Fetal Heart Rate Monitor + US Biophysical profile |
|  |  |  |  |  | UVV size and fluence |
|  |  |  |  |  | UA PI |
|  |  |  |  |  | Fetal Growth follow up |
|  |  |  |  |  | DV PI |

15. 2 For Moderate grade

| Not at all | Once in two weeks | Once a week | Twice a week | Once a day |  |
| --- | --- | --- | --- | --- | --- |
|  |  |  |  |  | Fetal Heart Rate Monitor + US Biophysical profile |
|  |  |  |  |  | UVV size and fluence |
|  |  |  |  |  | UA PI |
|  |  |  |  |  | Fetal Growth follow up |
|  |  |  |  |  | DV PI |

15. 3 For Severe grade

| Not at all | Once in two weeks | Once a week | Twice a week | Once a day |  |
| --- | --- | --- | --- | --- | --- |
|  |  |  |  |  | Fetal Heart Rate Monitor + US Biophysical profile |
|  |  |  |  |  | UVV size and fluence |
|  |  |  |  |  | UA PI |
|  |  |  |  |  | Fetal Growth follow up |
|  |  |  |  |  | DV PI |

16. Does any UVV require inpatient monitoring?
☐ Yes, all UVV ☐ Only moderate/severe ☐ Only severe ☐ No hospitalization required

# Timing of Delivery

17. At what gestational age would you deliver pregnancies with mild, moderate, and severe UVV?

17.1 For Mild grade
(Options: 24–28 / 29–30 / 30–31 / 32–33 / 34–35 / 36 / 37 / 38 / 39 weeks / No early delivery)

17.2 For Moderate grade
(Options: 24–28 / 29–30 / 30–31 / 32–33 / 34–35 / 36 / 37 / 38 / 39 weeks / No early delivery)

17. 3 For Severe grade
(Options: 24–28 / 29–30 / 30–31 / 32–33 / 34–35 / 36 / 37 / 38 / 39 weeks / No early delivery)

# Mode of Delivery

18. Is UVV an indication for cesarean delivery?
☐ Yes, all cases ☐ Only moderate/severe ☐ Only severe ☐ Not an indication

# Additional Comments

19. _______________________________________________
